# Case Report: Cardiac Multiple Thrombus and Pulmonary Embolism Associated With Mycoplasma Pneumonia Infection in a Child

**DOI:** 10.3389/fped.2022.959218

**Published:** 2022-07-18

**Authors:** Tongqiang Zhang, Jiafeng Zheng, Hongbo Wang, Yongsheng Xu, Jing Ning, Chunquan Cai

**Affiliations:** ^1^Department of Respiratory, Tianjin Children’s Hospital (Children’s Hospital of Tianjin University), Tianjin, China; ^2^Tianjin Institute of Pediatrics, Tianjin Key Laboratory of Birth Defects for Prevention and Treatment, Tianjin Children’s Hospital (Children’s Hospital of Tianjin University), Tianjin, China

**Keywords:** Mycoplasma pneumoniae, cardiac thrombus, pulmonary embolism, children, infection

## Abstract

Mycoplasma pneumoniae (MP) is a common pathogen of lower respiratory tract infection in children and adolescents. Some patients with MP infection are self-limiting, while with the increase of severe or refractory Mycoplasma pneumoniae pneumonia (MPP) in recent years, there is a great increase in reports of thromboembolism in multiple organs, including lung, brain, spleen, and peripheral arteries. Cardiac multiple thrombi and pulmonary embolism associated with MP infection have not been reported. The most effective treatment option for cardiac thrombus was surgical resection for fear of thrombus detachment and causing new thromboembolism. Herein, we present a patient with cardiac multiple thrombi and pulmonary embolism in MPP for the first time. In our case, the child recovered after conservative medical treatment, which provides a therapeutic option for children with cardiac multiple thrombi.

## Introduction

Mycoplasma pneumoniae (MP) is a common pathogen of lower respiratory tract infection in children and adolescents and has been known to cause various kinds of extrapulmonary manifestations including rash, vasculitis, liver function damage, nervous system sequelae, hemolytic anemia, pericarditis, and thrombosis (1–3). With the increasing cases of children with severe Mycoplasma pneumoniae pneumonia (MPP) in recent years, thromboembolism in various organs including lung, brain, spleen, and peripheral arteries has been reported (1, 4), while multiple intracardiac thrombosis was very rare. We report a previously healthy 8-year-old girl with severe pneumonia developed multiple cardiac thrombus and pulmonary thromboembolism, and etiological examination confirmed MP infection. After thrombolysis, anticoagulation, systemic application of corticosteroids and antibiotics, the prognosis was good. The aim of this study was to provide clinical experience for the diagnosis and treatment of such diseases.

## Case Description

**FIGURE 1:**
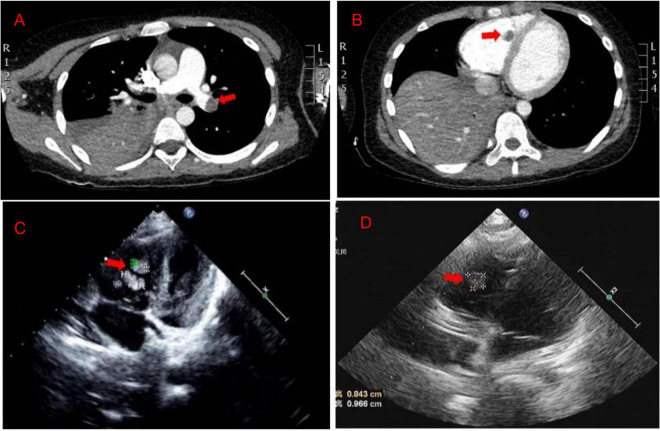
**(A,B)** The chest CT angiography showed a filling defect in the left lower pulmonary artery and right ventricle. **(C)** Echocardiography revealed multiple mass echos in the right ventricle (4 × 3 mm, 13 × 5 mm, and 9 × 5 mm) on day 3. **(D)** Echocardiography revealed mass echos in the right ventricle (8 × 13 mm) on day 12.

A previously healthy 8-year-old girl was referred to the Department of Respiratory at Tianjin Children’s Hospital (China) for 5 days of high fever and 2 days of violent coughing. The patient had no related histories of congenital metabolic disease, congenital heart disease, cardiovascular surgery, family thrombus, antiphospholipid antibody syndrome, systemic lupus erythematosus, dilated cardiomyopathy, nephrotic syndrome, inflammatory bowel disease, etc. During the course of the disease, there was no hemoptysis, fatigue, and syncope in the patient. A lung computed tomographic (CT) scan revealed a massive consolidation shadow in the right lung lower lobe and pleural effusion. Despite intravenous administration of ceftriaxone (80 mg/kg/day) and azithromycin (10 mg/kg/day) for 3 days at the local hospital, fever and respiratory status continued to deteriorate. Therefore, she was referred to our hospital on 30 July 2019. Evaluation of her cold agglutinin titer of MP-IgM revealed 1:160 while C-reactive protein level was >200 mg/L. She was administered intravenous latamoxef (80 mg/kg/day) combined with azithromycin (10 mg/kg/day) as anti-infection medications and methylprednisolone (2 mg/kg/day) as an anti-inflammatory medication. On day 2, elevated inflammatory marker and liver function levels were detected (neutrophil ratio 90%, ferritin 653 ng/L, lactic dehydrogenase 653 U/L, alanine aminotransferase 109 U/L, and aspartate aminotransferase 29 U/L). MP-DNA concentration in bronchoalveolar lavage fluid was 5.0 × 10^7^ copies/ml. There were no bacteria, fungus, or viruses (adenovirus, respiratory syncytial virus, influenza virus, rhinovirus, human metapneumovirus, Rhinovirus, and Epstein Barr virus) found in sputum, bronchoalveolar lavage, and blood. On the third day, she developed a sudden pain on the right side of her neck and dyspnea. Echocardiography (Figure 1C) revealed multiple mass echos in the right ventricle (4 × 3 mm, 13 × 5 mm, and 9 × 5 mm). There was no enlargement of the right ventricle and right atrium. Echocardiography showed ejection fraction was 77%, left ventricular short axis shortening was 45%, with normal ventricular wall motion and systolic function. The children had normal blood pressure, without enlargement of the liver and enlarged jugular vein. There was no galloping rhythm in heart auscultation. The myocardial enzymes and myocardial injury markers in the patient were also normal. Chest CT angiography showed a filling defect in the left lower pulmonary artery and right ventricle (Figures 1A,B). Coagulation analysis revealed elevated D-dimer levels (10 mg/L; normal reference range, 0–0.55 mg/L) and fibrinogen (FIB) (6.683 g/L; normal reference values, 2–4 g/L). Based on these findings, cardiac multiple thrombus and pulmonary thromboembolism was suspected (Table 1). This disease is life-threatening and usually treated by surgical intervention for fear of thrombus detachment and caused new thromboembolism. However, the patients’ parents opted for conservative medical treatment. Therefore, she was administered with intravenous urokinase (initial 4,400 IU/kg, 10 min, later 4,400 IU/kg/h, q12h, total 5 days) for thrombolysis, low molecular weight heparin calcium (200 IU/kg/d, q12h) for anticoagulation and aspirin (4 mg/kg/day) to inhibit platelet aggregation. The symptoms improved after thrombolysis for 9 h, and only two massive echoes (9 × 5 mm and 13 × 5 mm) were found in the echocardiogram after 11 h. We also explored the thrombophilia screen. Plasma protein C activity 152.3% (normal reference values, 70–140%), plasma protein S activity 46.85% (normal reference values, 70–123%), Antithrombin III activity increased by 132.2% (normal reference values, 75–125%). Anti-nuclear antibodies (ANAs) were positive, titer 1:100, karyotype: Golgi and granular type. Anticardiolipin (aCL) antibodies and anti-β 2-glycoprotein antibodies were both negative, while lupus anticoagulant (LA) was positive. There was no definite pathogenic mutation in the screening of thrombotic disease-related single gene genetic diseases.

**TABLE 1:** General information of MPP children.

The basic characteristics	Case
Age (year)	8
Gender	Female
Embolism position	Right ventricle, left Pulmonary arterial
Outside the lung damage	Liver damage
Hypoxemia	Yes
Thoracic puncture	Yes
Fiberoptic bronchoscopy	Yes
Length of hospital stay	50 days
Days of fever	9 days
Days of thrombus from onset	Hospital day 3
Days of thrombus disappear	3 months after leaving the hospital
Antibiotics before admission	Ceftriaxone sodium and azithromycin for 2 days
Antibiotics after admission	Cephalosporin,
Anti-inflammatory therapy	Methylprednisolone 6 mg/kg/d
Human immunoglobulin	
Thrombolytic therapy	Urokinase
Anticoagulant therapy	Sobilin, rivaroxaban, aspirin
Plasma protein C activity	152.3%(normal reference values, 70–140%)
plasma protein S activity	46.85%(normal reference values, 70–123%)
Anti-thrombin III activity increased	132.2%(normal reference values, 75–125%)
karyotype: Golgi type plus granulation; anticardiolipin antibody negative, anti-β 2-glycoprotein antibody	(-)

**FIGURE 2:**
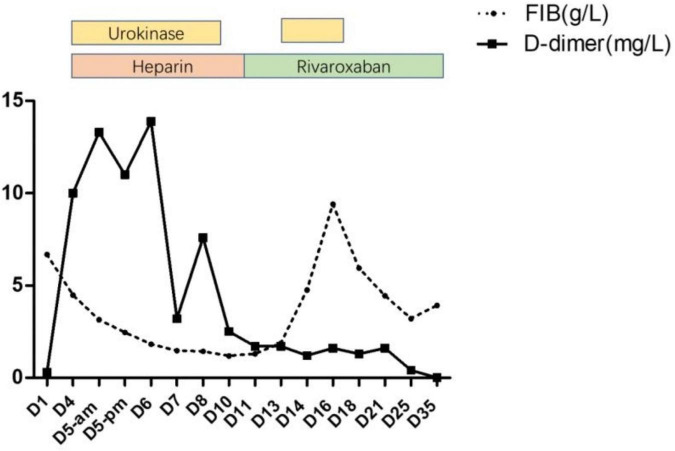
Changes of FIB, D-dimer before and after application of thrombolytic drugs.

After 10 days of hospitalization, her condition gradually stabilized and the filling defect of the left lower pulmonary artery was decreased by CTA in the lung. Administration of low molecular weight heparin calcium was changed to rivaroxaban (15 mg/qd) as the anticoagulation therapy. However, on day 12, the patient suddenly complained of pain in the right shoulder and intercostal region and fidgety. Echocardiography (Figure 1D) showed a right ventricular mass echo (8 mm × 13 mm). One of the right ventricular thrombus had disintegrated and caused a new pulmonary embolism. She was treated with urokinase (4,400 IU/kg/h, q12) for 3 days again (Figure 2), and rivaroxaban anticoagulation therapy was continued. On day 13, she did not complain of pain in the shoulder or intercostal region. Hospitalization was continued for 30 days and the echocardiography still showed a mass echo (about 6 mm × 11 mm) in the right ventricle. After which she was discharged and oral rivaroxaban administration was continued. After 2 and 3 months of follow-up, there was no cardiac thrombosis and pulmonary embolism respectively. In 2 years of follow-up, we found no recurrence of thrombosis in the heart and lungs.

## Discussion

In this study, we first reported a child who initially suffered from respiratory tract infection, pulmonary CT showed massive inflammatory changes, accompanied by pleural effusion. There was a significantly increased in the levels of blood inflammatory markers, liver function, and D-dimer. The child was previously healthy and had no history of cardiovascular disease, immune system disease, blood disease, external injury, or specific drug history. Multiple cardiac thrombosis and pulmonary thromboembolism were confirmed by chest CTA and echocardiography, and only MP infection was confirmed by etiological examination. After thrombolysis, anticoagulation, systemic application of corticosteroids and antibiotics, the prognosis was good.

In recent years, reports about thrombosis or thromboembolism in vessels related to MPP have gradually increased in the Chinese Mainland and abroad (4, 7, 8). However, cases of cardiac thrombosis in children with MP infection are rare. In 2006, Bakshi et al. first reported left atrial thrombosis in a 4-year-old child with MP infection (9). Li et al. found 2 cases of 6-year-old MPP with intracardiac thrombosis (10): 1 case with right atrial thrombosis 8 days after onset, and another case with right ventricular thrombosis 11 days later. There was also a 9-year-old patient with right ventricular thrombus 8 days after the onset of MPP symptoms (5). This study reports for the first time that children with severe MPP are complicated with multiple cardiac thrombi and pulmonary embolism 8 days after the onset of fever. Although the incidence of thrombosis varies, thrombus occurs within 8 days to 3 weeks after fever or cough (average 12 days) (5). The clinical manifestation of cardiac thrombus was not typical except for the manifestation of lung disease, and it was discovered by accident during the examination of cardiac ultrasound (10, 11). In this study, the children suddenly suffered neck pain, chest pain, dyspnea, and other clinical manifestations, which cannot be determined as the unique manifestation of cardiac thrombus, as may be the manifestation of pulmonary embolism. In this case, it was found that imaging examinations of respiratory and circulatory systems, such as lung CT and cardiac ultrasound, should be actively applied in case of severe MPP in school-age children. Among the reported cases, one of the 43 children with MPP complicated with thrombus had a history of allergic purpura, and the others did not mention a history of underlying diseases (4). The patient in our report also had no related histories of congenital metabolic disease, heart disease, cardiovascular surgery, family thrombus, nephrotic syndrome, etc. Therefore, children with severe MPP without underlying diseases should be alert to the occurrence of thrombosis.

The pathogenesis of MP infection complicated with thrombosis or embolism is complicated, including direct invasion, immune-mediated injury reaction, hypercoagulable or thrombotic state, vasculitis, and toxin injury. The common monitoring indexes of the hypercoagulable states include D-dimer and FIB. D-dimer is a specific degradation product of cross-linked fibrin, which reflects blood hypercoagulable state, intravascular thrombosis, and secondary fibrinolysis. Several reports of MPP complicated with pulmonary embolism in children showed that D-dimer was significantly higher than normal (12–14). According to a research on 43 children with MPP complicated with thrombosis, it was found that FIB and D-dimer reached the peak within 6–15 days after the onset of the disease, with concentrations of 4.5 ± 2.2 g/L and 11.1 ± 12.4 mg, respectively. Patients with >5.0 mg/L accounted for 58.1% and with >2.0 mg/L accounted for 93.0% (4). This case reported that the change of D-dimer was consistent with the above research. The D-dimer was 0.3 mg/L on admission. When multiple cardiac thrombi and pulmonary embolism occurred on the eighth day, the D-dimer increased significantly (10 mg/L). After thrombolytic therapy, the level of D-dimer temporarily increased to the peak (13.9 mg/L) and decreased gradually. Therefore, dynamic monitoring the levels of D-dimer is of great significance to the pediatrician. In addition, we also revealed the dynamic changes of fibrinogen. In our case report, the level of FIB was 6.683 mg/L on admission and decreased gradually after thrombolytic therapy. Surprisingly, the levels of FIB increased again on the 13th day (after the second thrombolysis for 1 day), reached the peak (9.402 g/L) on the 16th day, and then decreased gradually. Throughout the whole course of the disease, abnormal blood coagulation indexes may still occur even after active anticoagulation treatment, reminding pediatricians of the importance of dynamic monitoring of blood coagulation function to avoid the possibility of systemic bleeding due to excessive anticoagulation. At the same time, avoid anticoagulation not timely which leads to blood re-entering the hypercoagulable state.

Protein C, protein S, and Antithrombin III are important factors causing intravascular agglutination. When the activity of protein C and protein S decreases, it is easy to form a blood hypercoagulable state and promote thrombosis (15). In this case, the activity of protein S also decreased temporarily and returned to normal after recheck after 2 months. Graw-Panzer et al. reported a case of MP infection complicated with pulmonary and popliteal vein embolism and detected a temporary decrease in its protein S activity, suggesting that the blood hypercoagulable state may be promoted by affecting the decrease of protein S activity in children with MPP (13).

It has been reported that autoimmune inflammation caused by antiphospholipid antibodies (aCL, LA, and anti-β 2 glycoproteins) and ANAs play an important role in the process of thrombosis (16, 17). Liu et al. (4) analyzed 43 children with MPP complicated with thrombus in Beijing Children’s Hospital and found that the positive was 50% in ANAs, 60% in aCL antibodies, 64% in β2 glycoprotein-IgM, and 42.1% in LA. In this case, ANAs and LA were positive, which was consistent with the previous report. It is suggested that the transient hypercoagulable state caused by antiphospholipid antibodies and ANAs induced by MP infection may play an important role in the pathogenesis of Mycoplasma-associated thrombosis.

Echocardiography and CTA play an important role in the diagnosis and monitoring of cardiac thrombosis and pulmonary thromboembolism. Echocardiography can directly detect cardiac thrombosis and was widely used in the clinic because of its simple operation, with no trauma and radiation for the patients (18). However, if lack of vigilance for Mycoplasma infection-related cardiac thrombosis, it is easy to be misdiagnosed as a tumor or other diseases. It was reported that a 9-year-old child with MP infection showed right ventricular mass shadow by echocardiography. Because it was difficult to determine whether the mass shadow in the cardiac was a tumor, surgically was conducted for diagnosis and treatment of diseases. Pathological examination confirmed that the mass shadow was a relatively new fibrin thrombus with less white blood cells (5). Therefore, it should be combined with clinical manifestations, laboratory index, and other imaging findings to avoid missed diagnosis and misdiagnosis. The examination of CTA can quickly determine the location and degree of cardiac thrombus and pulmonary embolism (19), which is widely used in clinical practice. This child was diagnosed with pulmonary thromboembolism by chest CTA. In addition, there are three cases in which pleural effusion was detected in cardiac thrombosis patients after MP infection (10, 11). Our study also found pleural effusion in this case, therefore, more attention should be paid to children with severe MPP complicated with pleural effusion.

The treatment of MPP complicated by cardiac thrombus is rare and there is no unified standard. At present, it mainly includes anticoagulation therapy, thrombolytic therapy, and surgical thrombectomy. The choice of treatment depends on the patient’s clinical manifestation, embolus size, number, location, and hemodynamics. There was a 9-year-old patient with MPP who showed a right ventricular mass partially detached and almost floating in the cardiac by echocardiography, the patient underwent surgical thrombectomy (5). In another case complicated with right ventricular thrombus (17.0 mm × 9.3 mm) after anticoagulant therapy for 12 days, reexamination of echocardiography showed that there was no significant change in the thrombus, and surgical thrombectomy was performed (10). Our team recommended surgical intervention for fear of thrombus detachment and causing new thromboembolism. However, the patients’ parents refused and opted for conservative medical treatment.

Thrombolytic therapy is the best treatment for acute thromboembolism in adults; however, its application in children is still rarely reported. The main thrombolytic drugs were urokinase, streptokinase, and recombinant tissue plasminogen activator (20). There are few reports on urokinase treatment of MPP complicated with a thrombus in children. A 5-year-old boy with MPP complicated by popliteal artery embolism was reported in Korea. The popliteal artery blood flow recovered and the clinical symptoms improved after intra-arterial infusion of urokinase (21). There was also a report that a 5-year-old patient with a thrombus of the left atrium and right middle cerebral artery was treated with low molecular weight heparin and aspirin after 24 h of urokinase treatment, and the cardiac thrombus disappeared 9 days later (11). In this case, we performed urokinase thrombolysis twice, and one cardiac embolus fell off 11 h after the first thrombolysis. Heparin drugs (unfractionated heparin or low molecular weight heparin) and Vitamin K antagonists were used for anticoagulation treatment. Li et al. (10) reported that a case of 6-year-old MPP complicated with right atrial thrombus (7.5 mm × 4.0 mm) was initially treated with low molecular weight heparin for 7 days, followed by sequential oral warfarin for 2 months, and the intracardiac thrombus disappeared. In our report, the patient was admitted with low molecular weight heparin for 7 days, followed by oral Rivaroxaban for 3 months, cardiac thrombus and pulmonary embolism gradually disappeared, and the prognosis was good. A phase 3 clinical trial of Rivaroxaban in the treatment of acute venous thromboembolism in children reported that, compared with standard anticoagulants (low molecular weight heparin or Vitamin K antagonist), Rivaroxaban can reduce the probability of the thrombus recurrence without increasing the risk of bleeding (22). Ma et al. (23) reported a case of the thrombotic storm in children controlled by Rivaroxaban and there was no thrombotic recurrence and bleeding during follow-up. Rivaroxaban was also applied to tumor-associated venous thrombosis in a child for one month, and the left upper limb brachial vein thrombosis disappeared (24). The experience of using Rivaroxaban in the treatment of MPP complicated with cardiac thrombosis and pulmonary embolism has not been reported. This case provides thrombolysis and anticoagulation experience for the treatment of multiple cardiac thrombosis and pulmonary thromboembolism.

## Conclusion

We provide a rare case of MP infection complicated with cardiac multiple thrombi and pulmonary embolism in children. The aim of this study was to provide clinical experience for the diagnosis and treatment of such diseases.

## Data Availability Statement

The original contributions presented in this study are included in the article/supplementary material, further inquiries can be directed to the corresponding authors.

## Ethics Statement

Written informed consent was obtained from the individual(s) for the publication of any potentially identifiable images or data included in this article.

## Author Contributions

JN and CC: conception and design. YX: administrative support. TZ: provision of study patient. HW: collection and assembly of data. JZ: search literatures. All authors wrote the manuscript, contributed to the intellectual content of this manuscript, and approved the final manuscript as submitted.

## Conflict of Interest

The authors declare that the research was conducted in the absence of any commercial or financial relationships that could be construed as a potential conflict of interest.

## Publisher’s Note

All claims expressed in this article are solely those of the authors and do not necessarily represent those of their affiliated organizations, or those of the publisher, the editors and the reviewers. Any product that may be evaluated in this article, or claim that may be made by its manufacturer, is not guaranteed or endorsed by the publisher.
